# Ready for phase 5 - current status of ethnobiology in Southeast Asia

**DOI:** 10.1186/s13002-015-0005-7

**Published:** 2015-02-22

**Authors:** Syafitri Hidayati, F Merlin Franco, Rainer W Bussmann

**Affiliations:** Curtin Sarawak Research Institute, Curtin University Sarawak Malaysia, CDT 250, 98009 Miri, Sarawak Malaysia; William L. Brown Center, Missouri Botanical Garden, P.O. Box 299, St. Louis, MO 63166-0299 USA

**Keywords:** Biocultural diversity, Socio-ecological systems, Traditional knowledge, Indigenous people, Biodiversity, Ethnobotany, Ethics, Review, Five phases

## Abstract

**Background:**

Southeast Asia is known for its rich linguistic, cultural and biological diversity. While ethnobiology in the west has benefitted greatly from intellectual and methodological advances over the last decades, the status of Southeast Asian ethnobiology is largely unknown. This study aims to provide an analysis of the current status of ethnobiology in Southeast Asia and outlines possibilities for future advancements.

**Methods:**

We accessed papers cited in the Scopus and Web of Science databases for the period of 1960 to 2014 using the current as well as previous names of the 11 Southeast Asian countries and various disciplines of ethnobiology as key words. We juxtaposed the number of publications from each country against its number of indigenous groups and languages, to see if ethnobiology research has addressed this full spectrum of ethnical diversity. The available data for the last ten years was analysed according to the five phases concept to understand the nature of studies dominating Southeast Asian ethnobiology.

**Results and conclusions:**

A total number of 312 publications were recorded in the databases for the period 1960–2014. Indonesia ranks highest (93 studies), followed by Thailand (68), Malaysia (58) Philippines (42), Vietnam (31), Laos (29), and other Southeast Asian countries (44). A strong correlation was found between the number of publications for each country, the number of indigenous groups, and the number of endangered languages. Comparing the data available for the period 2005–2009 with 2010–2014, we found a strong increase in the number of phase 5 publications. However, papers with bioprospecting focus were also on the rise, especially in Malaysia. Our study indicates that ethnobiologists still need to realise the full potential of the Biocultural Diversity of Southeast Asia, and that there is a strong need to focus more on socially relevant research.

## Introduction

Ethnobotany as an academic discipline surfaced in the 19th century. Initially named ‘Aboriginal Botany’ by Powers in 1874 [1], the discipline received its widely accepted name from Harshberger in 1895 [2,3]. True to its name and a research ethics dominated by colonial principles, ethnobotanical research primarily dealt with the relationship between ethnic communities and plants until 1944, when Castetter coined the term ‘ethnobiology’ to signify the use of plants and animals by ‘primitive’ people [4]. Today, ethnobiology encompasses a wide range of sub-disciplines such as ethnozoology, ethnoecology, ethnopharmacology, ethnomedicine, ethnomycology, and ethnoveterinary, with often-amorphous boundaries [5]. Even before the emergence of ethnobotany and ethnobiology as disciplines, various societies and individuals explored the relationship between humans and plants and animals [6]. The development of ethnobiology in fact started with the compilation of ancient medicinal knowledge, e.g. in Greece, Egypt and Asia [7,8], and 1874 only marks the beginning of western style academic research. Old systems of medicine are believed to be the written compilation of contemporary folk knowledge, and thus folk knowledge can be considered as the precursor of all traditional medicinal systems. For instance, traditional Indian medicinal systems such as the Ayurvedha, Siddha and Yoga, acknowledge folk medicinal knowledge as the root source of information [9]. Later, Renaissance texts such as the meticulously compiled Hortus Malabaricus were also documenting the folk botanical knowledge [10].

In the late 19th century, ethnobiology research was mostly spearheaded by ethnographers and linguists [11], while interdisciplinary approaches only gained prominence in the 20th century. Clement [12] categorises the development of ethnobiology as a discipline from the late 19th century onwards into three phases: (1) the preclassical period (1860–1899), when terms such as ethnobotany and ethnozoology were first coined, (2) the classical period (1950-1980s), when ethnobiologists started emphasizing more on ‘emic’ and (3) the post classical period (1990s), marked by the emergence of real collaborations between western scientists and indigenous people. Hunn [13] later expanded these three phases into four - Phase 1 (1895–1950): documentation of ‘useful’ plants and animals beginning with the coining of the word ‘ethnobotany’ in 1895; Phase 2 (1954-1970s): the phase of ‘cognitive ethnobiology’ or ‘ethnoscience’ where cognitive psychology and linguistics played an important role, with Berlin’s work on folk biological classification system as a remarkable achievement of this phase [14,15]; Phase 3 (1970s-1980s): emergence of ethnobiology with an ecological focus, with ethnoecological concepts of Traditional Ecological/Environmental Knowledge, Indigenous Knowledge, Traditional Knowledge and Wisdom, Local Ecological/Environmental Knowledge, and Socio-ecological systems emerging [16]; Phase 4 (1990s): with the development of collaborative research, equitably involving both the researcher and the community, with emphasis on community rights [17]. The principles of Prior Informed Consent (PIC) and Intellectual Property Rights (IPR) became essential components of ethnobiological research. By 1992, the first Code of Conduct was issued by the International Society of Ethnobiology, and amended in 2001, 2006, and 2008 [18]. One important development of phase 4 was the development of biocultural concepts [19-21]. Another concept that is similar to the biocultural diversity, but uses a ‘systems’ based approach is the concept of socio-ecological systems [22]. Later, various researchers explored the links between linguistic, cultural and biological diversity to give shape to the concept of biocultural diversity [20,23-28].

Researchers have called for a phase 5 of ethnobiology with increased networking among researchers of various discipline, to face the challenges of rapid ecological change and shifting political economies [29]. According to Wolverton [30], the Phase 5 of ethnobiology needs to cross traditional academic boundaries, focusing on solving problems related to contemporary environmental and cultural crises. If phase 4 was about collaboration between researchers and communities, then phase 5 needs to focus on inter-disciplinary collaboration. While the idea of phase 5 is gradually shaping up, the concern for vanishing cultures and biodiversity, and the capability of interdisciplinary research to meet such challenges has already led to the development of the concept of Biocultural Diversity (BCD); thus, considering the increasing understanding of the language-knowledge-culture-biodiversity matrix and the collaborations happening between linguists-anthropologists-ethnobiologists, phase 5 could very well be the phase of BCD. Ethnobiological research is however still often undertaken by the western scientists. Of an estimated 300 million indigenous people in the world, 50-60% live in Asia [31,32]. Southeast Asia in particular is a very heterogeneous region, characterised by enormous ethnic, linguistic and biological diversity. Yet, ethnobiological research in Southeast Asia has yet to mirror this diversity. In this paper, we present a review of the status ethnobiology studies undertaken so far in Southeast Asia and the possible scope lying ahead.

### Methodology

**Figure 1:**
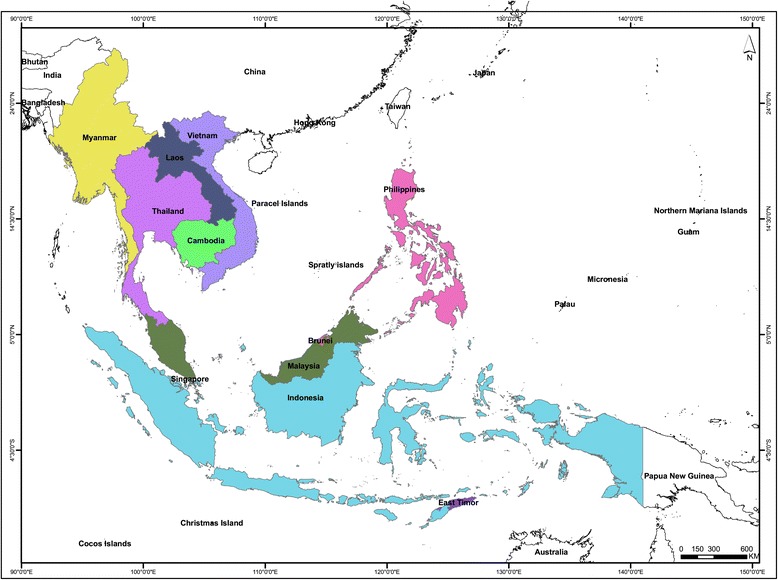
**Map of Southeast Asia.** Source: DIVA-GIS [37].

Our study is inspired by a recent review by Albuquerque *et al*. on the status of ethnobiological research in Latin America [33]. Publications cited in the Scopus and Web of Science databases for the period of 1960 to 2014 were identified using the previous as well as current names of the 11 Southeast Asian countries and various disciplines of ethnobiology as key words in all possible combinations. The following eleven countries were chosen on the basis of the definition of Southeast Asia given by Winzeler [34]: Thailand, Indonesia, Malaysia, Myanmar, Brunei, Vietnam, Singapore, Philippines, Laos, Cambodia, and Timor Leste (Figure 1). Owing to their history of colonisation, some of the present day Southeast Asian countries were either part of other countries or known by different names. Hence, their pre-colonial names such as Tanah Melayu (Brunei, Sarawak, Sabah), Burma/Birma (Myanmar), Kampuchea (Cambodia), East Timor (Timor Leste), Hindia Belanda (Indonesia), Siam (Thailand) and Shonanto (Singapore) were also used as keyword. Ethnobiology disciplines or areas related to ethnobiology included are: ethnoecology, ethnobotany, ethnozoology, ethnopharmacology, ethnomedicine, ethnoveterinary, ethnomycology, biocultural, traditional knowledge, traditional medicine, socio-ecological, as well as ethnobiology. We decided to include the term socio-ecology also, as the concept recognises the role of traditional knowledge in determining the relationship between communities and ecosystems [22]. According to a study carried out by Falagas *et al*. with PubMed, Scopus, Web of Science, and Google Scholar, the combination of Scopus and Web of Science (WOS) can be applied efficiently to track academic studies [35]. WOS is focused on citation analysis while PubMed is an important resource in biomedical research. However, Scopus covers an expanded spectrum of journals than WOS or PubMed. Although Google Scholar as an indexing tool has gained popularity and is relatively easier to operate, the results usually include articles from journals that are not peer-reviewed. Moreover, Google Scholar’s index also include books, as well as unreliable ‘grey literature’ such as website articles, drafts and pre-prints of submitted articles, abstracts, conference papers, and scientific reports [35,36]. Although there are numerous ethnobiology studies published in the form of books both in English and regional languages, the present study does not deal with them due severe constraints faced in translating and locating them.

We considered a publication to be of ethnobiological in nature when it dealt with the relationship between human beings and any natural resource; studies that were purely pharmacological in nature were excluded. After identifying the studies, information such as country where the research was conducted, year of publishing, area of study (ethnobiology, ethnopharmacology, etc.), nature of the study according to the concept of five phases [13,30] and number of international collaborations were extracted. Publications that mainly intend to catalogue the plants/animals used by a group of people were considered as phase 1 in nature, those dealing with cognitive ethnobiology/linguistic ethnobiology were considered of phase 2 in nature, publications on ethnoecology were considered as phase 3, those resulting from collaboration between the communities and researchers with emphasis on community rights, PIC and IPR were considered as phase 4 and those dealing with BCD and socio-ecological systems were considered as phase 5. The number of publications from each country was juxtaposed against the number of indigenous groups and endangered languages in each country, to see if ethnobiological research has managed to realise its full potential on the biocultural front. The phase wise data for the last ten years was analysed according to the five phases concept [13,30], to see i) if the trend is applicable to Southeast Asia ii) the nature of studies dominating Southeast Asian ethnobiology.

## Results and discussion

### Ethnobiology in Southeast Asia- country wise analysis

**Figure 2:**
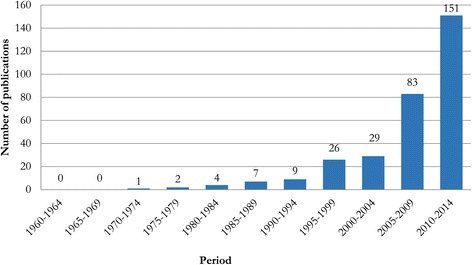
**Number of ethnobiology studies in Southeast Asia published during 1960–2014.**

Our query returned more than 3000 publications for the period of 1960–2014, from which, a total number of 312 publications were selected on the basis of the pre-defined criteria. Of these 312 publications, at least 102 resulted from international collaborations. The data shows that the number of annual ethnobiological publications has grown gradually from only one in 1972 to 151 publications in 2010–2014; No publications were recorded in the databases for the period of 1960–1969. Figure 2 gives a quick idea of the increasing number of ethnobiology publications. According to Cotton [3], ethnobotanical studies began in Asia in 1981 when the Society of Ethnobotanists, India began publishing their journal. However, our study shows that the history of ethnobiology research in Southeast Asia actually begins much earlier, going back at least to 1972 with the publication of “The Asian species of *Strychnos*. Part I. *Strychnos* as a source of the drug *Lignum colubrinum* (snake-wood)” by Bisset [38]. This publication reports the use of *Strychnos* for medicinal purposes in India, Indonesia and Sri Lanka. If we consider the coining of the term ‘Aboriginal Botany’ in 1874 as the beginning point of academic ethnobiology, then it has taken almost a century for ethnobiology to get started in Southeast Asia; like in Europe and elsewhere, Southeast Asian ethnobiologists initially focused on the medicinal uses of plants. The second study recorded from Southeast Asia was conducted by Ellen, who pioneered ethnobiological research in Indonesia, in Central Seram, eastern Indonesia, in collaboration with Nuaulu, a small group of hunters, collectors, and cultivators [39]. The first studies recorded in the databases for other countries were, Philippines: Neumann and Lauro [40]; Malaysia: Houghton [41]; Thailand: Anderson [42]; Vietnam: Stephenson [43]; Brunei: Bernstein [44]; Laos: Rao *et al*. [45]; Myanmar: Fujisaka *et al.* [46]; East Timor: Collins *et al*. [47]; Cambodia: Eisenbruch [48] Singapore: Loh [49]. However, considering the fact our study takes into account only those papers recorded in the Scopus and Web of Science databases, the possibility of non-indexed publications pre-dating the above publications cannot be ruled out.

Our analysis of studies done in each country shows that Indonesia ranks highest with 93 studies, followed by Thailand (68), Malaysia (58) Philippines (42), Vietnam (31), Laos (29), and other Southeast Asian countries (44) (Table 1). Singapore recorded the least number of publications (04); as an urban and immigration destination, Singapore does not have native or indigenous people [50], but the ethnic diversity brought about by three major ethnic groups (Chinese, Malay, and Indian) offers plenty of scope for ethnobiological studies. Moreover, ethnobiology is not an exclusive study of indigenous communities, and an increasing number of ethnobiology research is being carried out in urban ecosystems [51-53]. Indonesia, Thailand, and Malaysia have emerged as the three major centres of ethnobiology in Southeast Asia; these countries are also home to a great number of indigenous communities. Indonesia has the highest number of indigenous people in Southeast Asia, with at least 365 officially recognized ethnic groups [54]. Given that the data used was compiled from various sources, it has to be considered cautiously. Being home to a large number of indigenous groups, Indonesia, Thailand, Malaysia, Laos, Vietnam, and Myanmar possess a high number of endangered languages that face the risk of extinction, and it should be noted here that various researchers have shown a positive correlation between linguistic, ethnic and biological diversity [28,55]. We compared the country wise data for publications with the number of indigenous communities and languages in each country (Table 1). The correlation between the number of publications for each country, the number of indigenous groups (r = 0.73), the number of total languages (r = 0.76), and the number of endangered languages (r = 0.77) was found to be strongly significant. This indicates that countries with a high number of ethnic communities and endangered languages have generally returned high number of ethnobiological studies, and also points to the tremendous potential for improvement, by working with communities and policy makers to document and salvage the cultural and linguistic heritage before they are lost.

**Table 1:** **Country wise data for languages, ethnic groups and number of publications**

**Country**	**Total Languages**	**Indigenous Languages**	**Total Endangered languages**	**Vulnerable**	**Definitely endangered**	**Severely endangered**	**Critically endangered**	**Extinct**	**Ethnic groups**	**TOTAL publications**
**Brunei Darussalam**	16	14	0	-	-	-	-	-	8	8
**Cambodia**	25	22	19	1	1	8	9	0	24	13
**East Timor**	19	19	6	4	1	0	1	0	16	6
**Indonesia**	718	705	148	56	30	19	31	12	365	93
**Laos**	92	86	32	3	22	5	2	0	49	29
**Malaysia**	101	93	26	5	9	9	3	0	94	58
**Myanmar**	120	116	28	3	7	8	6	4	135	13
**Philippines**	196	180	15	2	3	3	3	4	110	42
**Singapore**	32	24	0	-	-	-	-	-	0	4
**Thailand**	84	71	26	2	15	4	4	1	34	68
**Vietnam**	110	108	27	5	15	4	3	0	53	31

Source: [54,56,57].

### Southeast Asian ethnobiology- moving towards phase 5?

**Figure 3:**
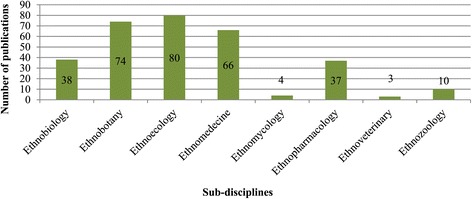
**Discipline wise analysis of ethnobiology studies during 1960–2014.**

**Figure 4:**
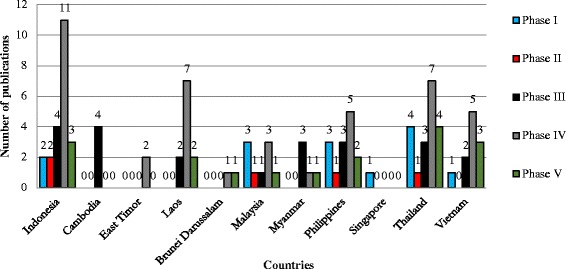
**Phase wise analysis of Southeast Asian ethnobiology for the period 2005–2009.**

**Figure 5:**
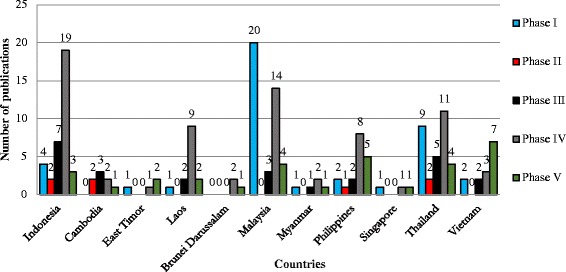
**Phase wise analysis of Southeast Asian ethnobiology for the period 2010–2014.**

Our analysis for the nature of ethnobiology publications from Southeast Asia from 1960 to 2014 shows that 64 publications could be classified as phase 1, 19 as phase 2, 49 as phase 3, 122 as phase 4 and 58 as phase 5. Sub-discipline wise, ethnoecology has received special attention with 80 publications, followed by ethnobotany (74), ethnomedicine (66), ethnopharmacology (37), ethnozoology (10), ethnomycology (04), ethnoveterinary (03). Thirty eight publications were categorised as ethnobiology as they dealt with more than one sub-discipline (Figure 3). Data for the past ten years (2005–2014) shows that the last decade has seen a manifold increase in the number of papers (Figures 4, 5). Although in 2005–2009, research from Indonesia, Malaysia, Philippines, and Vietnam focussed on the documentation and cataloguing of biodiversity traditionally used by indigenous people, majority of such studies involved international collaboration which can be considered as phase 4. During this period, Singapore produced only one publication.

Publications about research in Malaysia, Philippines, and Thailand on ‘cognitive ethnobiology’ or ‘ethnoscience’ (phase 2) were lower when compared with the total publications of phase 1. One of the publications from Mynmar [58] was about hydropower and sustainability, tagged with ethnoecology as key word (phase 3).

During the period 2010–2014, the total publication rate increased. Interestingly, Malaysia’s publication count of phase 1 papers increased from three in 2005–2009 to 20 during 2010–2014, indicating the increasing emphasis on bioprospecting (Figures 4,5). However, it is encouraging to note that 21 publications from Malaysia during the same period were of phase 3, 4 and phase 5 in nature. This trend is also observed in data from Thailand, Philippines and Indonesia, which were producing ethnobiology publications in all five phases during that time. In these countries, research of phase 4 type, especially studies concerned with indigenous people’s rights, dominated. It is encouraging to note that researchers from Southeast Asian countries are putting greater emphasis on conforming to ethical guidelines (of their institution, government, or codes of ethics), establishing prior consent (written, oral, and direct agreement between researcher and community), and acknowledging indigenous people as knowledge holders by establishing benefit sharing agreements with the community. All 11 countries had ethnobiology studies involving international collaboration (Phase 4), giving importance to ethical issues including Prior Informed Consent (PIC), recognition of indigenous peoples right over knowledge and resources [18,59]. Seventy-two publications could be considered as phase 4; although some of these focused on cataloguing, they also considered knowledge holders’ rights by obtaining prior informed consent and were guided by various code of ethics. In addition, they were also collaborating with local researchers [33].

During 2005–2014, eight out of the 11 countries had shown interest in the biocultural diversity and socio-ecological systems. In their paper, Wyndham *et al*. [29] underline the importance to make ethnobiology relevant to today’s biocultural crisis. This message has clearly reached Southeast Asia. Comparing the data available for the periods of 2005–2009 and 2010–2014, we found an increase in the number of publications of phase 5. However, given the tremendous Biocultural Diversity of Southeast Asia, there is a wide scope for increasing the number of publications. Deforestation, mining, land rights, loss of agrobiodiversity, change of agricultural patterns, climate change impacts for example have turned out to be the major problems faced both by indigenous communities and the environment. Swidden agriculture, the most common form of agriculture practiced throughout Southeast Asia, is yet to be fully understood, while at the same time around 163 million people in East Asia are said to be undernourished [60,61]. Ethnobiologists could help to ensure better nutritional security by promoting agrobiodiversity and diversification of food sources. Laos, Malaysia, Cambodia, and Indonesia have lost millions of hectares of forest and land to oil palm and rubber plantations, and large scale mining operations [54]. These activities clearly endanger both cultural and biological diversity. The occupation of native lands for these purposes is leading to increasing social conflicts. Ethnobiologists need to work with communities and governments to mitigate the impact of deforestation by fostering community based conservation [62,63]. Native land rights are becoming an important issue, especially in Malaysia where there is tremendous scope for researchers to work with both communities and governments to mitigate conflicts. Yet, most of the ethnobiology studies from Malaysia still deal with documenting natural resources and traditional knowledge on plant or animal uses, while land issues have so far been sidelined. One reason for this could be the ‘politically-safe’ nature of bioprospecting, which, unlike deforestation and land issues, does not attract controversy in the region. A single lone study by Vaz and Agama, discussed the role of indigenous and community-conserved areas in Sabah, Malaysia [64]. Favourable government policies are an essential factor for empowering indigenous communities; such policies are also essential for undertaking ethnobiology research [65].

## Conclusions

Though Southeast Asian ethnobiology only started after more than a century after the dawn of ethnobiology, it is encouraging to note that the region has kept up with the pace of developments happening in the field. In the future, the international research community should especially work with researchers from Cambodia, East Timor, and Singapore to fill large existing gaps in ethnobiology studies. Of the three countries, Singapore is a developed country with a robust economy, which would facilitate local mobilisation of resources. While working with researchers from least developed countries such as Myanmar, Laos, Cambodia and East Timor, collaborations need to emphasize local capacity building and mobilisation of external financial resources. Although our analysis shows that Southeast Asian ethnobiology has not reflected the existing biocultural diversity adequately, it also indicates that there is a great potential for ethnobiologists, to help conserving the rich biocultural heritage of Southeast Asia.
